# The last 10 years: any changes in perceptions of the seriousness of alcohol, cannabis, and substance use in Canada?

**DOI:** 10.1186/s13011-019-0243-0

**Published:** 2019-12-05

**Authors:** John A. Cunningham, Anja Koski-Jännes

**Affiliations:** 1 Centre for Addiction and Mental Health & University of Toronto, 33 Russell St., Toronto, ON M5S 2S1 Canada; 2 Tampere University, Tampere, Finland

**Keywords:** Alcohol, Tobacco, Medical drugs, Gambling, Illicit drugs, Attitudes, Epidemiological survey

## Abstract

**Background:**

Over the last decade, there have been a number of changes in the Canadian landscape - the deconstruction of alcohol policy in some provinces, the legalization of cannabis, increased availability of gambling options, and the increase in opioid use and its associated problems. Have there been concomitant changes in societal images of addictions?

**Methods:**

A general population survey on societal images of addictions was conducted in multiple countries in 2008 - Finland, Sweden, Canada (Canadian sample size: *N* = 864; 40% response rate), and part of Russia (St Petersburg). We repeated the same survey in 2018 in Canada (*N* = 813; response rate = 23%). The survey assessed perceptions of the seriousness of different issues to society - including items about alcohol, tobacco, marijuana, gambling, misuse of medical drugs, and drugs like amphetamine, cocaine, or heroin - among other items (e.g., pollution, violent crime, prostitution).

**Results:**

There were increases in perceptions of the seriousness of misuse of medical drugs (*p* = .001), of illicit drugs (*p* = .005), ratings of the seriousness of cannabis use (*p* = .02), and a decrease in ratings of gambling as a social problem (*p* = .04). Ratings of the seriousness of alcohol and tobacco as social problems did not display significant changes over time (*p* > .05).

**Conclusions:**

There has been some variation in societal perceptions of the seriousness of different addictions. Increases in perceptions of the seriousness of misusing medical drugs and the use of illicit drugs may reflect increases in societal concerns about opioid use and its associated problems. Despite substantial changes in alcohol control policies, the legalization of cannabis, and the increased availability of options for gambling, there appears to be very little associated change in societal perceptions regarding these addictive behaviours.

## Background

In Canada, as with many other countries, there have been noticeable changes over the last decade in governmental policies regarding alcohol and other substance use, and also, some changes in patterns of their consumption. Some provinces in Canada (the level at which most alcohol legislations are implemented) have continued to dismantle their control policies, allowing alcohol to be sold in more locations (e.g., the province of Ontario now allows sales of beer in selected supermarkets), and over longer periods each day [1]. In addition, cannabis has now been legalized after almost two decades of moves to allow medical use and to decriminalize possession and consumption of small quantities of the drug [2]. There are ongoing evaluations of the impact of this policy change on increases in prevalence of cannabis use [3]. Further, opportunities for gambling are becoming increasingly available (e.g., online gambling, sports betting) [4]. Finally, Canada has followed the United States (US) on its path through increased use of opioids and concomitant increases in opioid overdose deaths [5].

How does the general public react to these changes? Are there variations over time in people’s attitudes and beliefs about different addictive behaviours that coincide with these structural changes in how substances are controlled, and in patterns of consumption and consequences? And, if there are changes in beliefs, why could it matter?

The images people hold of the nature of addictions are related to their views about the ways addiction problems should be solved (e.g., is treatment needed and abstinence required?, [6–8]). These beliefs could lead addicted individuals to experience stigma and other barriers to recovery from their problems [9, 10]. In addition, societal beliefs about the nature of addictions have policy implications insofar as the allocation of resources reflects societal views about the nature of such problems and their cures (e.g., a ‘war’ on drugs approaches, the criminalization of cannabis consumption in some countries, or the establishment of treatment facilities).

A decade ago, a cross-national series of studies sought to explore the general public’s beliefs about the nature of addictions, their severity, and their cure in more depth. Parallel surveys were conducted in Finland, Sweden, St. Petersburg (Russia), and Canada. Primarily, the general public’s views about a number of addictions were assessed and contrasted with their views on other societal problems (e.g., violent crime, pollution, social inequality). A series of publications were produced that helped to bring into focus people’s images of addiction by comparing and contrasting these views across different countries with similar geographic and climatic characteristics [11–14]. Notable in these comparisons were the extent to which representations of ‘hard’ drugs cluster with views of criminality and badness [15]. In contrast, cannabis use displayed substantial variations, with less negative views expressed by participants from Canada than those from Finland and Sweden (which may reflect the higher prevalence of cannabis use in Canada compared to Sweden and Finland; separate cannabis items were not asked in Russia) [16]. Alcohol was regarded as one of society’s most serious problems in Finland, perhaps reflecting the increased problems experienced at that time due to reductions in taxes (and alcohol prices) resulting from European Union (EU) membership (alcohol consumption was regarded as somewhere in the middle relative to rating of other societal problems by the other countries) [15].

Another method of highlighting societal views on addictions is to explore whether they change in a country over time. To this purpose, the same general population survey was conducted in Canada a decade after the original was administered (2008 and 2018). The occasion was stimulated by the legalization of cannabis. However, given changes over the last decade in other substances, and in availability of gambling, we sought to examine changes in attitudes for all these addictive behaviours (rather than just for cannabis) on the extent to which they are viewed as societal problems.

## Methods

Two random digit dialing telephone surveys were conducted of Canadians, 18 years and older, the first in 2008 (*N* = 864) and the second in 2018 (*N* = 813). Interviews were conducted in English or French. Each telephone number in the sample received up to 14 contact attempt calls. Calls were scheduled during the day, evenings and weekends. Respondents were asked a series of questions starting with 15 items that asked them to rate on a scale from 1 (not at all serious) to 10 (extremely serious), how serious they thought a number of different issues were to society – see Table 2 for a list of issues asked about. The surveys concluded with a series of demographic items.

Analyses consisted of bivariate comparisons for each item between the 2008 and 2018 surveys. In addition, two principal components analyses with varimax rotation were conducted, one for the 2008 and the other for 2018 survey, in order to compare the component structures of these 15 items. Results are presented as weighted values to be representative of the Canadian adult population (sample sizes are presented as unweighted values).

## Results

The response rate for the 2008 survey was 41%. Using the same response rate computation for the 2018 survey as the first survey, the landline sample had a response rate of 30%, the cell phone response rate was 10% and the weighted average of the two was 23%. Table 1 displays the demographic characteristics of the two surveys. In 2018, the mean [SD] age of the sample was higher than in 2008 (49.3 [17.9] vs 46.4 [17.4]; t = 2.84, 1626 df, *p* = .001), participants were more likely to have some post-secondary education (71.9% vs 66.5%; Fisher’s exact test, *p* = .02), less likely to have a household income of less than CAN$30,000 (11.0% vs 17.5%; Fisher’s exact test, p = .001), and less likely to be full/part time employed (60.8% vs 66.1%, *p* = .02).

**Table 1:** Demographic characteristics

	2008	2018	*p*
	(n = 864)	(n = 813)	
Mean (SD) Age	46.4 (17.4)	49.3 (17.9)	.001
% Male	49.4	49.0	N.S.
% Married/Common Law	65.8	64.1	N.S.
% Some post-secondary	66.5	71.9	.02
% Household income < $30,000	17.5	11.0	.001
% Full/part time employed	66.1	60.8	.02

*N.S.* not significant, *p* > .05

**Fig. 1:**
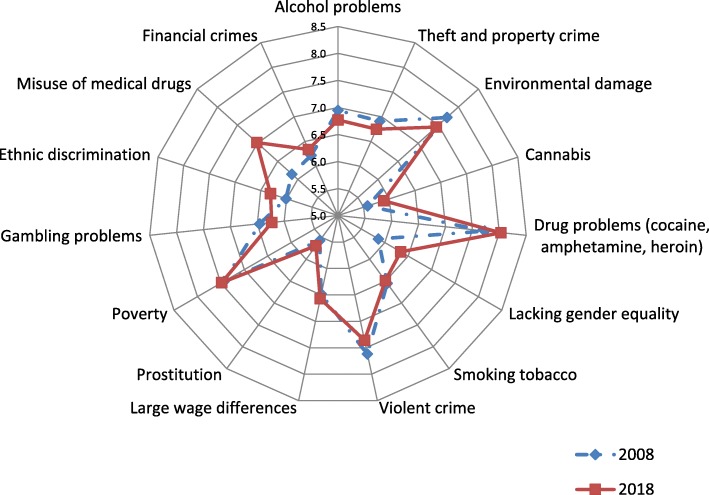
Do you think the following are serious problems for our society? (1 = not at all serious; 10 = extremely serious)

Figure 1 displays a radar diagram of participants’ ratings of the “seriousness of different social problems for our society.” In absolute terms, the societal problems rated as most serious in Canada remain largely unchanged, with drug problems (cocaine, amphetamine, and heroin), environment damage, violent crime, and poverty ranked as the most serious concerns. For the addictive behaviours, there was some variation between 2008 and 2018. While ratings of the seriousness of alcohol and tobacco as societal problems did not change significantly over time (*p* > .05), there was an increase in ratings of the seriousness of misuse of medical drugs (7.0 [2.1] vs 6.2 [2.4]; t = 7.6, 1582 df, *p* = .001, Cohen’s d = 0.38), some increases in ratings of drug problems (8.0 [2.1] vs 7.7 [2.2]; t = 2.82, 1634 df, *p* = .005, d = 0.14) and cannabis use (5.9 [2.7] vs 5.6 [2.6]; t = 2.3, 1599 df, *p* = .02, d = 0.12), and a reduction in ratings of the seriousness of gambling as a social problem (6.2 [2.2] vs 6.4 [2.4]; t = 2.03, 1603 df, *p* = .042, d = 0.10). For the other nine societal problems asked about, four displayed changes from 2008 to 2018, with increases in concerns about lacking gender equality (6.3 [2.3] vs 5.8 [2.4]; t = 4.1, 1621 df, *p* = .001, d = 0.20) and ethnic segregation (6.3 [2.4] vs 6.0 [2.5]; t = 246, 1576 df, *p* = .014, d = 0.12), and decreases in concern about environmental damage and violent crime (respectively: 7.4 [2.1] vs 7.7 [2.0]; t = 2.63, 1666 df, *p* = .009, d = 0.13; 7.4 [2.3] vs 7.6 [2.3]; t = 2.32, 1626 df, *p* = .021, d = 0.11).

Table 2 displays the results of two principal components analyses with varimax rotation (for 2008 and 2018 respectively). The component structure of the ratings of the seriousness of different problems for society was very similar in 2008 and 2018. In both surveys, two factors were identified. All addictive behaviours loaded onto factor 1, whereas societal inequality issues received the highest loadings on factor 2. Smoking tobacco, gambling problems, and misuse of medical drugs also loading onto factor 2 in 2008 at a level of 0.4 or greater. In 2018, only misuse of medical drugs loaded onto factor 2, with the remaining addictive behaviours solely loading onto factor 1 (at a level of 0.4 or greater). The other item loading exclusively on factor 1 was theft and property crime, although a substantial number of the other social problems loaded onto both factors.

**Table 2:** Rotated principle components matrix of societal problems, factor loadings

	2008		2018	
	1	2	1	2
Alcohol problems	**0.687**	0.196	**0.635**	0.183
Theft and property crime	**0.654**	0.251	**0.657**	0.281
Environmental damage	0.220	**0.547**	0.160	**0.620**
Cannabis	**0.743**	−0.001	**0.718**	−0.011
Drug problems (cocaine, amphetamine, heroin)	**0.782**	0.174	**0.771**	0.098
Lacking gender equality	0.163	**0.710**	0.132	**0.666**
Smoking tobacco	**0.458**	**0.417**	**0.588**	0.218
Violent crime	**0.530**	**0.550**	**0.604**	**0.433**
Large wage differences	0.015	**0.711**	0.080	**0.655**
Prostitution	**0.629**	**0.484**	**0.565**	**0.495**
Poverty	0.265	**0.794**	0.272	**0.722**
Gambling problems	**0.573**	**0.459**	**0.632**	0.379
Ethnic discrimination	0.346	**0.673**	0.192	**0.732**
Misuse of medical drugs	**0.568**	**0.455**	**0.528**	**0.482**
Financial crimes	**0.509**	**0.514**	0.396	**0.623**
Initial eigenvalues	6.7	1.3	6.2	1.5
Varimax rotation; total explained variation	44.8	8.8	41.2	9.8

Bold data indicate a factor loading of 0.4 of greater

## Discussion

Between 2008 and 2018, there was some variation in ratings of the seriousness of different addictive behaviours as societal problems. Misuse of medical drugs displayed an increase in ratings of seriousness, perhaps reflecting the ongoing increased incidence of opioid overdose deaths and their media coverage [17, 18]. Drug problems and use of cannabis also displayed some increases in levels of concern. For drug problems, it is possible that this change is also the result of coverage of the opioid problem, as uptake of heroin and abuse of opioids is now intertwined in Canada (and the US) [19]. Increases in ratings of the seriousness of cannabis as a societal problem was unexpected (at least by the first author) based on the assumption that the increasing availability of cannabis in the past decade would have been mirrored by ratings in its decreasing seriousness as a societal problem. However, the 2018 survey was intentionally conducted around the time of legalization (half in the months before and half during the months after), and it is possible that participants in the survey were expressing some small increase in levels of concern about Cannabis, compared to participants in 2008, because it was unknown what would happen when Cannabis was legalized (e.g., would there be dramatic increases in amount of use?). Repetition of this survey in several years would be valuable to assess any sustained changes in ratings of cannabis as a societal problem.

Of the other societal issues assessed, the most substantial was the increase in concerns regarding gender inequality as a societal problem. This probably reflects the slowly growing support for female rights issues in society [20–22]. Variations in some other societal issues were also observed, with increased concerns about ethnic segregation and some decrease in ratings of concern about environmental damage and violent crime (although, also notable, concerns about environmental damage and violent crime remain two of the societal problems with the highest ratings as concerns).

Ratings of seriousness of alcohol as a societal problem did not display a significant difference (*p* > .05) between the two time points. This is despite a fairly consistent dismantling of alcohol control policies designed to reduce the harms associated with alcohol consumption across most provinces in Canada between 2008 and 2018. Any consequences of such dismantling may not be apparent to the general public though, as most participants will not live in locations next to bars with longer opening hours (and there appears to be some pleasure voiced that is associated with increased ease of accessing alcohol). Similarly, for gambling, there was some decrease in ratings of concern, despite increases in availability of options for gambling (e.g., online gambling). It is interesting that, although gambling is a government controlled activity in Canada [4], and there are various advertisements to ‘gamble responsibly,’ the majority of media (articles and advertisements) are of gambling as a fun activity [23, 24]. This presentation of gambling as a largely harmless activity may reflect its ratings of the seriousness of this activity as a societal problem. Finally, ratings of smoking tobacco remained similar. One of the largest changes in the tobacco control landscape over the last decade will have been increases in use of e-cigarettes [25, 26]. Unfortunately, the original survey in 2008 (and the 2018 survey, which was deliberately identical) did not contain items about e-cigarettes.

When considered in the context of the other societal problems asked about, only drug problems ranked highly along with environmental damage, violent crime, and poverty. This is despite alcohol causing significant harms to others (and to the drinker), and tobacco use remaining the number one contributor to preventable death [27]. Alcohol and tobacco use as harms to society appear to receive relatively little media coverage in comparison to opioid overdoses, despite both substances causing considerably more harm to society. Perhaps, as Blomqvist has suggested [11], perceptions of the seriousness of a societal problem have as much (or more) to do with the familiarity (or the legal status) of the activity than the actual level of danger and harm associated with it [28].

Another means of examining societal beliefs about the seriousness of different addictive behaviours is to explore the extent to which people covary their rating of each behaviour with those of other societal problems. Principal components analyses of both the 2008 survey and the 2018 survey displayed very similar structures. Perhaps meaningful is that smoking tobacco and gambling problems, while loading onto both factors in 2008, only loaded onto factor 1 in 2018 (sharing it with the other addictive behaviours and with theft and property crimes). One interpretation is that people are more clearly aligning smoking tobacco and gambling with addictions problems now than they were 10 years ago. Misuse of medical drugs continues to share loadings on both factors.

Both surveys had poor response rates, leading to concerns regarding the representativeness of the responses on these surveys to those of the general population of Canadians [29]. While not unique to this project, response rates this low lead to justifiable concerns about what segment of society is being surveyed. This concern is perhaps partially reduced in the current project because the intent is to look at changes in attitudes over time (rather than examining absolute ratings at one time point) and every attempt was made to make the surveys as similar as possible (i.e., the items were identical and administered in the same order). However, despite weighting of the survey data to approximate the characteristics of the Canadian population, there were differences in the demographic characteristics of the two samples (e.g., age) that cannot be accounted for by actual changes in the characteristics of the Canadian population at large [30]. Further, a conservative approach to analyzing this data would be to have adjusted the significance level based on the number of bivariate comparisons conducted (e.g., Bonferroni adjustment). This would have led to the interpretation of changes in ratings of the seriousness of cannabis and of gambling as not reaching significance for these two activities. However, we have chosen a more exploratory approach to these analyses and did not adjust for the number of comparisons made.

## Conclusion

In the last ten years in Canada, there appears to have been increased ratings of the seriousness of the misuse of medical drugs and illicit drugs, and some small increase in concerns regarding cannabis use. Repetition of these ratings in several years has merit in order to track societal views that may be associated with changes in patterns of use and legislation governing addictive behaviours.

## Data Availability

Available from the corresponding author on reasonable request.

## Abbreviations

CAN$: Canadian dollars
d: Cohen’s d
EU: European Union
US: United States
